# A Quantitative Perspective of Alpha-Synuclein Dynamics – Why Numbers Matter

**DOI:** 10.3389/fnsyn.2021.753462

**Published:** 2021-10-22

**Authors:** Christian G. Specht

**Affiliations:** Diseases and Hormones of the Nervous System (DHNS), Inserm, Université Paris-Saclay, Paris, France

**Keywords:** fluorescence recovery after photobleaching (FRAP), quantitative neurobiology, green fluorescent protein (GFP), gene dosage, liquid-liquid phase separation (LLPS), Parkinson’s disease (PD), Lewy body (LB)

## Abstract

The function of synapses depends on spatially and temporally controlled molecular interactions between synaptic components that can be described in terms of copy numbers, binding affinities, and diffusion properties. To understand the functional role of a given synaptic protein, it is therefore crucial to quantitatively characterise its biophysical behaviour in its native cellular environment. Single molecule localisation microscopy (SMLM) is ideally suited to obtain quantitative information about synaptic proteins on the nanometre scale. Molecule counting of recombinant proteins tagged with genetically encoded fluorophores offers a means to determine their absolute copy numbers at synapses due to the known stoichiometry of the labelling. As a consequence of its high spatial precision, SMLM also yields accurate quantitative measurements of molecule concentrations. In addition, live imaging of fluorescently tagged proteins at synapses can reveal diffusion dynamics and local binding properties of behaving proteins under normal conditions or during pathological processes. In this perspective, it is argued that the detailed structural information provided by super-resolution imaging can be harnessed to gain new quantitative information about the organisation and dynamics of synaptic components *in cellula*. To illustrate this point, I discuss the concentration-dependent aggregation of α-synuclein in the axon and the concomitant changes in the dynamic equilibrium of α-synuclein at synapses in quantitative terms.

## Introduction

### Fluorescence Imaging and Quantitative Neurobiology

**FIGURE 1:**
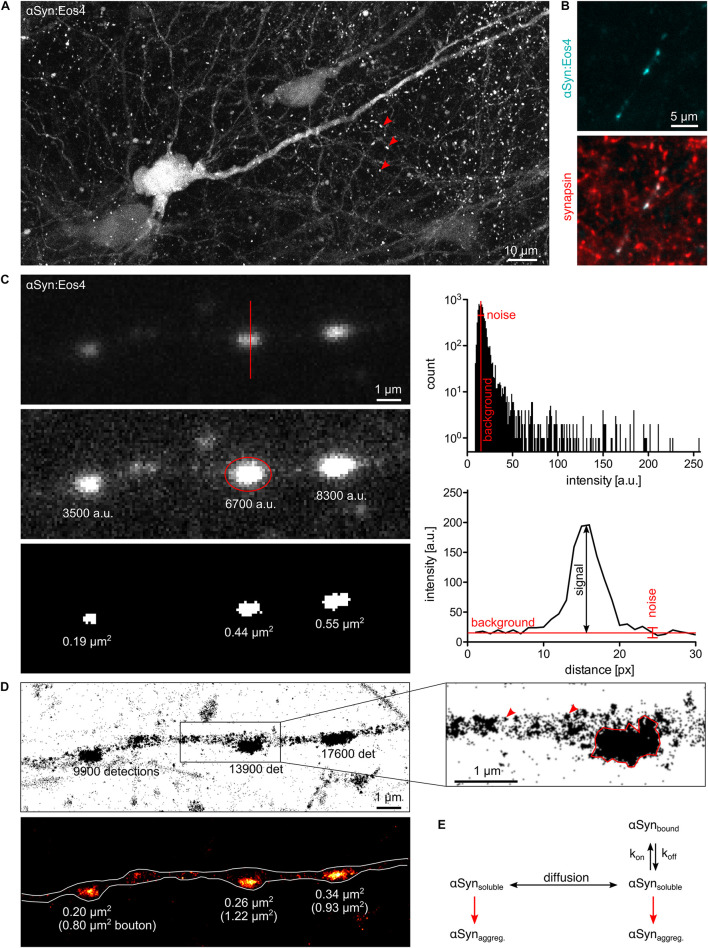
Quantitative fluorescence microscopy of α-synuclein. **(A)** Lentivirus-driven expression of αSyn:Eos4 in an organotypic hippocampal slice, visualised by confocal imaging using 488 nm illumination. The recombinant fusion protein is distributed throughout the somato-dendritic compartment of a pyramidal neuron and enriched in presynaptic terminals (red arrowheads). Scale: 10 μm. **(B)** Co-localisation of recombinant αSyn:Eos4 (low expressing construct, cyan) with endogenous synapsin I (immuno-labelling, red) at synapses in cultured cortical neurons. Scale: 5 μm. **(C)** Conventional fluorescence imaging of αSyn:Eos4 (low expressing construct) in a fixed cortical neuron (excitation 488 nm). Top panel: image with full dynamic intensity range (0–255 arbitrary units, a.u., frequency histogram). The red line denotes a trace through a synaptic bouton along which an intensity profile was measured. Middle panel: same image displayed with enhanced brightness for visibility. The values represent background-corrected integrated intensity readings of αSyn:Eos4 at individual boutons. Bottom panel: measurement of the apparent sizes of αSyn:Eos4 clusters in a binary image. Scale: 1 μm. **(D)** Single molecule super-resolution imaging of αSyn:Eos4 (561 nm laser excitation with photoconversion, 20,000 frames). Top: pointillist single molecule localisation microscopy (SMLM) image with the number of detections of each bouton. The zoom on the right shows that αSyn:Eos4 occupies a sub-region of the bouton, likely corresponding to the synaptic vesicle (SV) domain. Red arrowheads indicate clusters of detections arising from single mEos4b fluorophores. Bottom: rendered image showing that the high density domains of αSyn:Eos4 make up only 20–40% of the total area of the synapse. **(E)** Simplified model showing the dynamic equilibrium of α-synuclein at synapses. Increased expression or reduced synaptic binding at synapses raises the concentration of the soluble fraction of α-synuclein in the axon and promotes its aggregation throughout the neuron.

The quantitation of neurobiological experiments relies heavily on fluorescence microscopy. The strength of this approach lies in the fact that fluorescent signals can be measured accurately across a wide range of intensities (Figures 1A,B). Arguably, the most decisive breakthrough in quantitative imaging came with the discovery of green fluorescent protein (GFP) as a versatile fluorescent marker [reviewed in Piston et al. (1999)]. Using genetically encoded fluorophores fused to a protein of interest has the advantage that the labelling is specific and quantitative, resulting in a linear detection over a wide dynamic range. Furthermore, GFP is quite small and relatively inert, meaning that in many instances the tagging of proteins does not interfere with their localisation and/or function (e.g., Wang and Hazelrigg, 1994; Marshall et al., 1995). These qualities have driven the development of a growing palette of fluorescent proteins for specific applications including photoactivatable fluorescent proteins for super-resolution imaging and biosensors for functional imaging in living cells (Kim et al., 2021).

Almost any kind of fluorescence intensity measurement can be used to illustrate and compare differences in protein concentration between and within neurons. To qualify as *quantitative*, however, the data should meet a number of criteria. The fluorescence signals must be sufficiently bright to be distinguished from the background noise. The dynamic range should cover both the weakest signals above background as well as the brightest signals without reaching saturation (0–255 in an 8-bit image). Moreover, the acquisition should be conducted in the linear range, where pixel intensities increase in the same way as the amount of fluorescent proteins. If these conditions are met, the data provide accurate information about the *relative quantities* of fluorophores and by extension target proteins within a given cellular compartment (Figure 1C).

There are limits to the applicability of conventional fluorescence microscopy for quantitative neurobiology when it comes to the demarcation of the observed space. Diffusely distributed fluorophores within large compartments such as neuronal somata or thick dendrites produce greater signals than those within thin structures such as dendritic spines or axons. While confocal microscopy can prevent this effect to some extent by collecting only the emitted light from the focal plane (Figure 1A), the problem persists as the compartments get smaller. The underlying reason is that the point spread functions (PSF) of closely spaced fluorophores overlap as a result of the diffraction of light. In other words, the measurement of areas or volumes becomes meaningless when their size approaches the diffraction limit, as is the case for synaptic boutons, axons, or dendritic spines. Neither the size of these structures (in pixels or voxels) nor the fluorophore concentrations (in arbitrary units of intensity) can be determined accurately. Ultimately, the apparent size and signal intensity become inextricably linked and cannot be measured independently (Figure 1C). The only meaningful quantitative information that can be extracted under these conditions is the integrated intensity that reflects the total quantity of fluorophores within a given compartment, independent of the space occupied by the fluorophores.

### Single Molecule Localisation Microscopy and Absolute Quantification

Several super-resolution imaging approaches bypass the diffraction limit of fluorescence microscopy by essentially reducing the observed space and thus providing a more defined readout. Within the field of neurobiology, these approaches have begun to yield new structural insights that have changed our understanding of the internal organisation of neurons [reviewed in Werner et al. (2021)]. Some of the most remarkable discoveries to date are the identification of a periodic organisation of the actin cytoskeleton in axons and elsewhere in the neuron (Xu et al., 2013; Leterrier et al., 2015; Bär et al., 2016), or the *trans*-synaptic alignment of pre- and postsynaptic protein assemblies at excitatory and inhibitory synapses (Tang et al., 2016; Yang et al., 2021). Super-resolution imaging of Lewy bodies (LBs) has highlighted the presence of various organelles surrounding a crowded core containing α-synuclein, lipids, and fragmented membranes (Shahmoradian et al., 2019), which has led to a lively debate about the role of α-synuclein fibrillisation in the formation of LBs in Parkinson’s disease (PD; Lashuel, 2020; Ericsson et al., 2021).

A previously overlooked consequence of the gain in spatial resolution is that fluorescence intensity measurements can now be applied to more restricted sub-cellular compartments such as specific organelles, cytoskeletal elements, or, in the case of neurobiology, the postsynaptic density (PSD), and the presynaptic active zone (AZ). Since the estimation of the occupied space is more precise in super-resolution imaging, the concentration of a target protein in a specific compartment (the integrated fluorescence intensity divided by the area or volume) can be calculated quite accurately. This value has actual biological significance as it describes the relative enrichment of a protein at a given location, which ultimately reflects its diffusion properties and/or the strength of its molecular interactions. Hence the power of super-resolution imaging can be exploited to obtain new types of quantitative information.

Single molecule localisation microscopy (SMLM) is particularly well suited for quantitative analysis, since it not only achieves a localisation precision on the order of 10–20 nm, but also provides an exact quantitative readout in the form of single molecule detections (Lelek et al., 2021). This makes SMLM an inherently quantitative approach. The technique relies on the use of photoactivatable fluorophores that can be imaged sequentially rather than all at once. In this way, single fluorophore signals are temporally separated, which makes it possible to calculate the positions (and numbers) of the emitting molecules with great precision. Clustering algorithms have been repurposed to allow grouping of the detections into spatially and/or temporally defined subsets for quantitative analysis, including Ripley’s functions, DBSCAN, Voronoi tessellation and, more recently, graph-based approaches (Khater et al., 2020). The dynamic range of SMLM is theoretically unlimited from a true zero up to closely packed fluorophores. The sensitivity of SMLM is that of a single molecule (Figure 1D).

Single molecule localisation microscopy can even be used for *absolute quantification*, where the numbers of single molecule detections are converted into actual molecule numbers and packing densities (e.g., Maynard et al., 2021). Different approaches have been developed, generally involving some kind of internal calibration standard that can be extrapolated to clusters of detections arising from larger protein complexes or unknown structures (Wu et al., 2020). SMLM-based molecule counting is best performed with genetically encoded photoactivatable fluorophores, because this ensures the complete labelling of the target proteins, in particular when using a knock-in animal model. Quantitative SMLM analysis of endogenous proteins is also possible using immuno-labelling with reversibly blinking organic dyes (STORM). However, antibody labelling is notoriously non-linear, and these experiments are generally restricted to fixed samples (Lelek et al., 2021).

### Why Numbers Matter: α-Synuclein Dosage and Parkinson’s Disease

In many cases, a simple qualitative comparison of signal intensities may be sufficient to describe a biological effect. What then are the advantages of a fully quantitative approach? The strongest arguments for quantitative imaging are that (1) many biological phenomena are concentration dependent, (2) the relevance of a change in protein distribution is best evaluated on a linear scale, (3) quantitative data can be directly compared between different laboratories and experimental approaches, and (4) biophysical models rely on quantitative parameters to describe biological phenomena in mathematical terms. The need for quantitative information is exemplified by the presynaptic protein α-synuclein, because we do not yet fully understand many of the processes that underlie its dynamic behaviour within cells. In particular, the pathophysiology of α-synuclein is a uniquely quantitative problem.

Strongly enriched in presynaptic boutons (Figures 1A,B), α-synuclein is associated with synaptic vesicles (SV; Clayton and George, 1999) due to their lipid composition and curvature (Davidson et al., 1998). Other possible binding partners of α-synuclein include lipid rafts (Fortin et al., 2004), VAMP2 (Burré et al., 2010), as well as synapsin III (Zaltieri et al., 2015) and synapsin Ia (Atias et al., 2019). Based on the multiplicity of its molecular interactions hundreds of putative functions of α-synuclein have been proposed, as critically discussed by Vladimir Uversky (2017). Judging from the fact that the deficiency of α-synuclein and its paralogs β- and γ-synuclein does not result in overt phenotypes (Abeliovich et al., 2000; Chen et al., 2002; Connor-Robson et al., 2016), it is likely that α-synuclein plays a modulatory role in SV cycling that can be compensated by other presynaptic components. What makes α-synuclein one of the most studied macromolecules is that it plays a decisive role in PD and other neurodegenerative diseases referred to as synucleinopathies (Goedert and Spillantini, 1998). The first evidence linking α-synuclein to the pathophysiology of PD was the discovery of α-synuclein as the main protein component of LBs (Spillantini et al., 1997). Several point mutations in the *SNCA* gene that increase the propensity of α-synuclein to aggregate were identified in inherited cases of early onset PD (e.g., Polymeropoulos et al., 1997; Kruger et al., 1998). Certain conformations of wildtype and mutant α-synuclein produce β-sheeted fibrils (Iwai et al., 1995; Conway et al., 1998; El-Agnaf et al., 1998; Narhi et al., 1999) that first appear in the axons and eventually condensate as LBs in the somata of affected neurons (Volpicelli-Daley et al., 2011).

Overexpression of α-synuclein as a result of gene duplication or triplication is also associated with familial PD (Singleton et al., 2003; Chartier-Harlin et al., 2004; Ibáñez et al., 2004), indicating that the tendency of α-synuclein to aggregate is concentration dependent. It has recently been demonstrated that the formation of intracellular aggregates of α-synuclein in response to seeding of exogenous fibrils is more pronounced in cultured hippocampal neurons that express high endogenous levels of α-synuclein than in other neuronal populations (Courte et al., 2020). Since nucleation-dependent polymerisation processes are concentration and time-dependent and are strongly affected by the reaction conditions (temperature, pH, and buffer composition) (Hashimoto et al., 1998; Wood et al., 1999), understanding α-synuclein toxicity in neurons requires a quantitative in-cell approach.

### A Quantitative Approach to α-Synuclein Dynamics

As argued above, fluorescence microscopy offers a direct, quantitative view of α-synuclein distribution both in fixed and live neurons (Figure 1C). GFP-tagged α-synuclein accumulates at presynaptic locations similarly to the endogenous protein, suggesting that the fluorophore does not interfere with lipid binding (Specht et al., 2005; Caputo et al., 2020). Since the fusion of a small protein of 140 amino acid residues with a fluorescent protein of 250 residues could impair its function, the development of alternative tagging strategies is desirable. Nonetheless, the fact that the subcellular distribution α-synuclein is preserved justifies the use of genetically encoded fluorophores to study the protein dynamics of α-synuclein in living neurons.

A defining feature of α-synuclein is its exceptional mobility. Fluorescence recovery after photobleaching (FRAP) shows that GFP-tagged α-synuclein moves rapidly in the soma and the axon (Spinelli et al., 2014), probably in the form of freely diffusing monomers. At least two dynamic states of α-synuclein were identified at synapses, a fast component similar to the one in the axon, as well as a slower component that exchanges with a time constant of 2–3 min, pointing to the transient interaction of α-synuclein with synaptic binding sites (Spinelli et al., 2014). Occupancy of these binding sites depends on the strength of the molecular interactions, the concentration of free (soluble) α-synuclein and its diffusion in the axon, which creates a dynamic equilibrium between free and reversibly bound proteins. Interestingly, the mobility of α-synuclein also shapes its likely functional behaviour at synapses. In response to presynaptic activity, α-synuclein dissociates from the synaptic binding sites and is temporarily dispersed in the neighbouring axon, a property that it shares with other vesicle associated proteins such as synapsin (Fortin et al., 2005).

Although time-lapse imaging accurately describes the diffusion of α-synuclein at steady state or out of equilibrium, the interpretation of the data is complicated by the low spatial resolution of conventional fluorescence microscopy. The small diameter of axons and the small volume of presynaptic terminals present morphological constraints on diffusion that need to be taken into account. This is shown by the difference in the effective exchange rates of soluble α-synuclein measured in the soma and in the axon (Spinelli et al., 2014). Another consequence of the low spatial resolution is that the diffusion properties of molecules in spatially separated sub-domains cannot be studied independently, making it difficult to attribute the different kinetic states in FRAP experiments (Reshetniak et al., 2020). In particular, there is a distinct lack of information about actual fluorophore numbers, concentrations, and molecule fluxes of α-synuclein between the axon and the synapse. In classical FRAP experiments, fluorescence intensities are usually normalised to correct for differences in the initial intensity of synaptic puncta. Normalisation and the calculation of averages means that information about absolute molecule quantities is often disregarded. Since the occupancy of synaptic binding sites is dependent on the concentration of free α-synuclein, overexpression can saturate the binding sites, which may be partly to blame for conflicting experimental results (Fortin et al., 2005; Spinelli et al., 2014; Reshetniak et al., 2020; Weston et al., 2021). Another problem is that FRAP can induce phototoxicity and/or crosslinking (Lippincott-Schwartz et al., 2003), as suggested by the fact that most synaptic proteins display a significant immobile fraction, irrespective of their dynamic properties (Reshetniak et al., 2020). The detection of a stable component does therefore not necessarily prove the existence of aggregated α-synuclein at the synapse as has been suggested (Spinelli et al., 2014; Weston et al., 2021).

In contrast, the high spatial resolution of SMLM makes it possible to measure detection densities within defined axonal compartments. Quantitative single molecule imaging can thus give access to several relevant biophysical parameters. For example, the number of available binding sites of α-synuclein at synapses probably scales with the number of SVs. Molecule counting can yield copy numbers and absolute concentrations of α-synuclein. According to previous estimates, α-synuclein is very abundant, with about of 20–70 copies per SV and about 6,500 copies per bouton (Wilhelm et al., 2014; Fakhree et al., 2016). These values are likely to vary sharply between different neuronal cells and types of synapses. The affinity of α-synuclein for its synaptic binding sites is reflected in the steep concentration gradient between the synaptic and extrasynaptic α-synuclein populations. Single molecule localisation microscopy images of cortical neurons expressing low levels of recombinant α-synuclein tagged with the photoconvertible fluorophore mEos4b (αSyn:Eos4) show the enrichment of α-synuclein in synaptic boutons (Figure 1D). Most αSyn:Eos4 detections are concentrated in a sub-region of the bouton that probably corresponds to the SV domain. The concentration elsewhere in the bouton is much lower, to the point that clusters of detections from single molecules are visible and the notion of concentration itself becomes ill defined. The fact that the concentration of α-synuclein outside the SV domain is similar to that in the axon shaft suggests that this volume contains freely diffusing α-synuclein as observed by FRAP (Spinelli et al., 2014). Single molecule tracking can provide further information about diffusion and binding of α-synuclein at synapses, taking into account the existence of different diffusive states (e.g., Laurent et al., 2019; Verdier et al., 2021). If the association of α-synuclein with SVs gives rise to oligomers as suggested by Burré and colleagues (Burré et al., 2014), a degree of cooperativity of binding may be expected. An alternative model suggests that α-synuclein is clustered together with synapsin and SVs in a liquid phase (Hoffmann et al., 2021), which would be governed by particular stoichiometries and modes of diffusion.

## Conclusion

### A Spatio-Temporal Model of α-Synuclein Aggregation

In addition to a possible functional role, the dynamic properties of α-synuclein have important implications for pathology. There is some debate about the toxicity of different species of α-synuclein. One theory has it that LBs themselves are relatively inert (Goldberg and Lansbury, 2000), and that intermediate, toxic species such as misfolded oligomers or proto-fibrils bind to different cellular targets, disrupting essential physiological processes [reviewed in Gracia et al. (2020)]. The existence of distinct fibrillar polymorphs that can trigger α-synuclein aggregation in neurons and exhibit different phenotypic profiles clearly demonstrates that fibrils play a central role in the prion-like propagation of α-synuclein toxicity (Peelaerts et al., 2015; Shrivastava et al., 2020). However, there is no consensus on the initial dysregulation of endogenous α-synuclein in the recipient neurons and whether the toxic aggregates are formed at the synapse itself as some studies suggest (e.g., Kramer and Schulz-Schaeffer, 2007; Spinelli et al., 2014) or elsewhere in the neuron. An alternative explanation is that the aggregation of α-synuclein in the axon leads to its depletion at synapses, and that the pathological process is initiated or at least exacerbated by a loss of function of α-synuclein (Collier et al., 2016; Ninkina et al., 2020).

Lipid binding of α-synuclein was shown to induce an α-helical conformation in the N-terminal two-thirds of the protein (Davidson et al., 1998). Interestingly, lipid binding and the stabilisation of the α-helical structure also reduces the tendency of α-synuclein to form fibrils *in vitro* (Zhu and Fink, 2003). Based on these findings it can be hypothesised that α-synuclein aggregation in neurons depends on the concentration of free α-synuclein rather than the bound fraction at synapses. If these considerations are correct, any condition that shifts the dynamic equilibrium towards free α-synuclein, such as overexpression or changes in the affinity for synaptic binding sites is expected to accelerate the aggregation process (Figure 1E). It may further be speculated that the aggregation can begin anywhere in the neuron, since the concentration of freely diffusing α-synuclein is probably uniform. This would also be true if α-synuclein aggregation is driven by liquid-liquid phase separation (LLPS; Ray et al., 2020). That said, nucleation probably depends on the transmission process of toxic α-synuclein aggregates between cells. If membrane binding and internalisation is a random process, most transmission events are likely to occur on axons, due to their large total surface area. If, on the other hand, the transmission of toxic forms of α-synuclein depends on a synapse-specific mechanism (e.g., Shrivastava et al., 2020), nucleation would preferentially occur at synapses.

Quantitative imaging can provide essential information that helps distinguish between these possibilities. The systematic quantification of α-synuclein expression can substantiate the relationship between protein concentrations in neurons and their susceptibility to pathogenic insults. Since the concentration of soluble α-synuclein can be measured in the soma, this could be easily accomplished with conventional fluorescence microscopy, using a recently developed knock-in mouse model expressing endogenous α-synuclein-GFP (Caputo et al., 2020). SMLM can add ultra-structural and quantitative information when it comes to the characterisation of small compartments such as axons and synaptic terminals, including absolute copy numbers and shifts in the occupancy of synaptic binding sites. Quantitative super-resolution imaging has thus an important role to play when concentration-dependent processes are investigated in diffraction limited domains, which could provide new insights into α-synuclein dynamics and toxicity.

## Data Availability Statement

The raw data supporting the conclusions of this article will be made available by the author without undue reservation.

## Author Contributions

CS: conception and writing.

## Conflict of Interest

The author declares that the research was conducted in the absence of any commercial or financial relationships that could be construed as a potential conflict of interest.

## Publisher’s Note

All claims expressed in this article are solely those of the authors and do not necessarily represent those of their affiliated organizations, or those of the publisher, the editors and the reviewers. Any product that may be evaluated in this article, or claim that may be made by its manufacturer, is not guaranteed or endorsed by the publisher.
